# Bacterial sealing ability of calcium silicate-based sealer for endodontic surgery: an in-vitro study

**DOI:** 10.1186/s12903-024-04309-3

**Published:** 2024-05-21

**Authors:** Mai M. Mansour, Sybel M. Moussa, Marwa A. Meheissen, Mahmoud R. Aboelseoud

**Affiliations:** 1 Conservative Dentistry Department, Faculty of Dentistry, Alexandria University, Alexandria, 21527 Egypt; 2 DMedical Microbiology & Immunology, Faculty of Medicine, Alexandria University, Alexandria, 21527 Egypt

**Keywords:** Bacterial leakage, Calcium silicate sealer, Endodontic surgery, MTA, Retrograde filling, Single cone technique, Apical resection, Confocal laser scanning microscopy

## Abstract

**Background:**

Apical surgery with standard retrograde maneuvers may be challenging in certain cases. Simplifying apical surgery to reduce operating time and streamline retrograde manipulation is an emerging need in clinical endodontics.

**Aim of the study:**

The aim of the study was to compare the bacterial sealing ability of a calcium silicate-based sealer with the single cone technique combined with root end resection only, and calcium silicate-based sealer as a retrograde filling versus MTA retrofilling, and to analyze bacterial viability using confocal laser scanning microscope (CLSM).

**Materials and methods:**

In this in vitro experimental study, 50 extracted human maxillary incisor teeth were instrumented and randomly divided into five groups: three experimental groups, a positive control group, and a negative control group (*n* = 10/group). In the experimental groups, the roots were obturated using the single cone technique (SCT) and a calcium silicate-based sealer. In group 1, the roots were resected 3 mm from the apex with no further retrograde preparation or filling. In groups 2 and 3, the roots were resected, retroprepared, and retrofilled with either a calcium silicate-based sealer or MTA, respectively. Group 4 (positive control) was filled with a single gutta-percha cone without any sealer. In group 5 (negative control), the canals were left empty, and the roots were sealed with wax and nail varnish. A bacterial leakage model using Enterococcus faecalis was employed to assess the sealing ability over a 30-day period, checking for turbidity and analyzing colony forming units (CFUs) per milliliter. Five specimens from each group were examined using CLSM for bacterial viability. Data for the bacterial sealing ability were statistically analyzed using chi-squared and Kruskal-Wallis tests.

**Results:**

The three experimental groups did not show significant differences in terms of bacterial leakage, or bacterial counts (CFUs) (*P* > 0.05). However, significant differences were observed when comparing the experimental groups to the positive control group. Notably, the calcium silicate-based sealer, when used as a retrofilling, yielded the best sealing ability. CLSM imaging revealed viable bacterial penetration in all the positive control group specimens while for the experimental groups, dead bacteria was the prominent feature seen.

**Conclusion:**

Within the limitations of this study, it could be concluded that the bacterial sealing ability of calcium silicate-based sealer with the single cone technique combined with root end resection only and calcium silicate-based sealer as a retrograde filling were comparable with MTA retrofilling during endodontic surgical procedures.

## Introduction

The primary objective of surgical endodontic treatment is to eliminate unhealthy tissues and untreated apical ramifications to prevent the infiltration of bacterial biofilm and their by-products into the surrounding periapical tissues [1]. Achieving a tight apical seal at the junction of the conventional retrograde cavity and the root-end filling is cruical for successufl treatment outcomes [2]. While several products have been introduced as retrofilling materials over the years, the search for an ideal material remains ongoing.

Since its introduction in the 1990s, mineral trioxide aggregate (MTA) has emerged as the gold standard for root end filling material, owing to its superior chemical and biological properties. Despite its widespread adoption, MTA has certain limitations. These include challenging handling properties, limited resistance to compression and flexion, a heavy metal content that may contribute to discoloration of the treated tooth, and a prolonged setting time that increases the risk of material displacement in a damp surgical environment [3, 4]. .

In modern endodontics practice, significant advancements in techniques, materials, and instruments have been developed to enhance the success rate of endodontic surgery. Despite these advancements, periapical surgery remains a challenging procedure due to various factors including difficult access, limited visibility, proximity of lesions to vital structures, challenges in achieving hemostasis, and the intricate nature of apical root canal anatomy [5, 6]. Thus, controlled retrograde treatments with standard retrograde cavity preparation and filling may not be feasible in all cases [7, 8]. Accordingly, there is a need for a simpler and more feasible approach.

Recently, newly developed calcium silicate-based root canal sealers have become increasingly popular due to their favorable biological and physicochemical characteristics, such as chemical stability, biocompatibility, antimicrobial properties, and the induction of osteogenic reactions [9]. In addition, these sealers exhibit self-sealing ability through the formation of hydroxyapatite (biomineralization) and directly bonding to dentin [10].

Among the avaliable obturation techniques, the single-cone obturation technique (SCT) is commonly advised for use with calcium silicate-based sealers. This recommendation is supported by several compelling reasons, including minimal shrinkage compared to other sealers [11, 12], the ability to penetrate and polymerize into dentinal tubules, forming a hybrid layer that enhances root canal filling retention and entombs any persistent bacteraia [13]. This penetration not only improves the sealer’s microscopic adaptability but also creates a micromechanical lock [14]. Additionally, the SCT is a straightforward technique with fewer potential technical errors. From a clinical perspective, using a calcium silicate-based sealer with the SCT and root end resection only, could be considered an optimal treatment option in specific cases to streamline periapical surgery. This approach can be advantageous provided it achieves a proper seal of the root end cavity.

Endodontic micro-surgery may shift towards a technique that involves root end resection only after filling with a single cone and calcium silicate sealer. This aligns with the growing popularity of minimally invasive endodontics, which aims to simplify the complexities associated with standard apical surgery. However, this novel technique has not been extensively investigated from a microbiological perspective.

Moreover, if a calcium silicate–based sealer can be easily injected and effectively adapted to the root-end, it has the potential to facilitate the surgical procedure by reducing the required time while maintaining a high-quality root end filling. Despite this, calcium silicate-based sealers have been primarily assessed in the context of orthograde non-surgical root canal treatments only. Notably, no previous studies have investigated the use of calcium silicate based sealer as a retrograde filling. Therefore, the aim of this study was to compare the bacterial sealing ability of a calcium silicate-based sealer with the single cone technique combined with root end resection only, and calcium silicate-based sealer as a retrograde filling versus MTA retrofilling. Moreover, Bacterial viability using confocal laser scanning microscope (CLSM) was assessed. The null hypothesis was that no significant differences in bacterial sealing abilities would be observed between the different tested techniques.

## Materials and methods

### Tooth selection and preparation

**Fig. 1:**
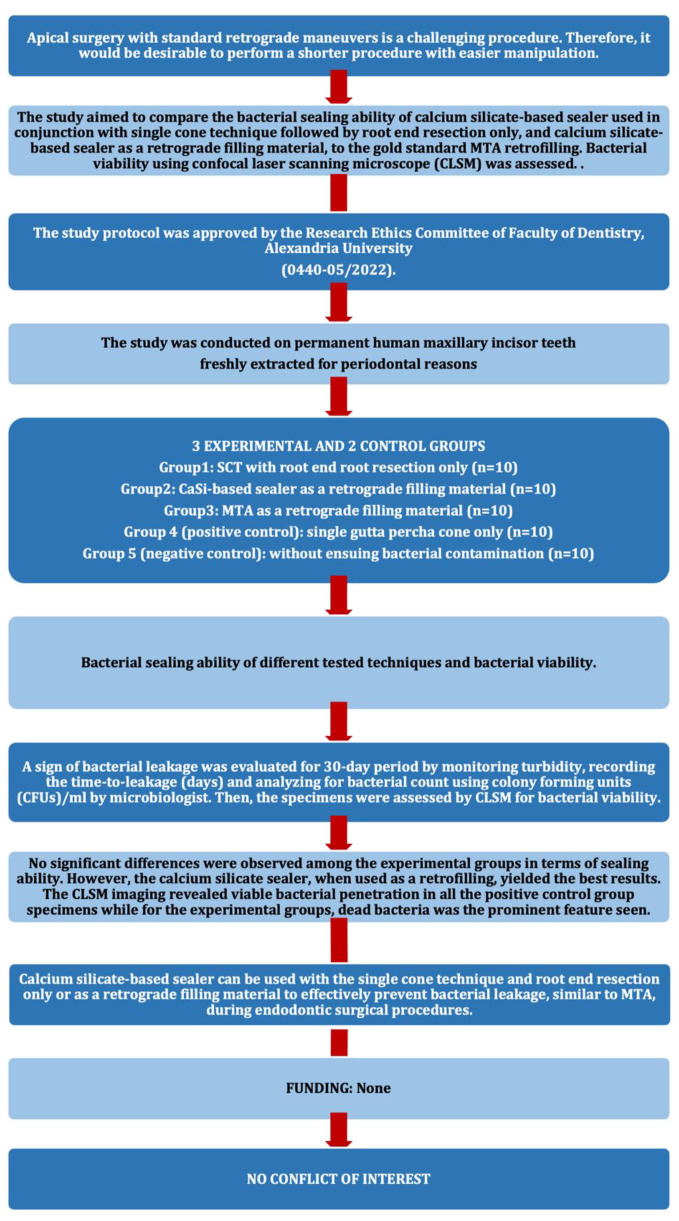
PRILE 2021 flow chart for the current study

This in vitro experimental study followed the Preferred Reporting Items for Laboratory studies in Endodontology (PRILE) guidelines, 2021 [15]. The PRILE 2021 flowchart is presented in (Fig. 1).

The study protocol received approval from the Research Ethics Committee of Faculty of Dentistry at Alexandria University (0440-05/2022).

Sample size calculations were performed using G*Power (Version 3.1.9.4). Sample size was calculated assuming 80% study power and 5% alpha error. Through a comparison of independent means, the minimum sample size was calculated to be 9 teeth per group, which was increased to 10 (×5 groups) to account for potential laboratory processing errors [16–18].

In this study, 50 permanent human maxillary incisor teeth freshly extracted due to periodontal reasons were collected. These teeth were carefully preserved in a 0.1% thymol solution. Only teeth with fully formed apices, a single root canal, and a single apical foramen confirmed through radiographic examination were included.

Teeth displaying any of the following characteristics were excluded from the study: developmental anomalies, root caries, cracks, fractures, perforation, previous root canal treatments, root resorption and canal curvatures exceeding 10 degrees [19].

The teeth were immersed in 5.25% sodium hypochlorite (NaOCl) solution (Chloraxid, Cerkamed, Poland) for 15 min to remove organic debris. Residual tissues were removed using a curette.

Next, the teeth were decoronated using a diamond disc (Keystone industries, Gibbstown, NJ, USA) to obtain specimens with a standardized length of 16 mm. The working length (WL) was determined by introducing a #15 stainless steel K-file (Mani, Nakanishi Inc., Tokyo, Japan) into the root canal until it became just visible at the apical foramen. One millimeter was then subtracted from this measurement [20]. Teeth with an apical diameter greater than 0.25 were excluded from this study.

### Root canal preparation

The root canals were instrumented at the WL up to size 40 with a 0.04 taper using Hyflex EDM rotary files (Coltene/Whaledent AG, Altstatten, Switzerland) at a speed of 500 rpm and a torque of 2.5 Ncm. Each rotary file was used to instrument three canals before being replaced. After each file, 3 mL of 2.5% NaOCl was used as an irrigant, and patency was maintained using a size #10 k-file. The smear layer was removed by rinsing with 3 mL of 17% EDTA (Dharma, Miami, Florida, USA) for 1 min, followed by 5 mL of 2.5% NaOCl for 3 min [20], and then 2 mL of sterile saline for 1 min to ensure complete removal of the NaOCl and EDTA [21]. The canals were then dried with paper points. All irrigating solutions delivered using a 30 G Max-i-Probe irrigating needle (Dentsply Rinn, Elgin, IL, USA), positioned 1 mm short of the WL.

The samples were subsequently autoclaved for sterilization. Each specimen was individually placed in a cryovial containing 500 µl of brain heart infusion (BHI) broth (HiMedia Laboratories Pvt. Ltd, India) and autoclaved at 121° C for 30 min. To verify the effectiveness of the sterilization process, the samples were incubated in their sealed tubes for 48 h at 37ºC, followed by subculturing from the vials onto blood agar.

At this stage, the prepared specimens were randomly assigned to three experimental groups and two control groups (*n* = 10 each) [22]. Specimens of the control groups were wrapped in moist gauze to prevent desiccation while waiting for the experimental groups to be obturated.

### Root canal obturation and root end procedures

After sterilization of specimens, for the three experimental groups, the root canals were flushed with 2 mL of sterile saline for 3 cycles, each lasting 20 s. Following the final rinse, the canals were dried using three sterile paper points, each of size 40/0.04 (Hyflex EDM paper points, Coltene/ Whaledent) [21].

Each canal was then trial-fitted with a gutta-percha cone size 40/0.04 taper (Hyflex EDM gutta-percha points, Coltene/ Whaledent), ensuring a tug-back at the WL. These cones were disinfected by immersing them in 2.5% NaOCl for 1 min, followed by a 1-minute immersion in 70% ethyl alcohol. Subsequently, the canals were filled using the single cone technique, employing a matched-taper cone and a calcium silicate-based sealer (NeoSEALER Flo Avalon Biomed, Houston, TX, USA). The obturation procedure was carried out in a sterile cabinet, following aseptic techniques.

The NeoSealer Flo was applied directly into the canal space using the included Flex Flo Tip™ from the kit. The Flex Flo tip was inserted into the coronal half of the canal, and while slowly expressing the sealer from the syringe, the tip was withdrawn from the canal. Next, a gutta-percha cone size 40.04 was coated with the sealer and then slowly inserted to the WL.

After the gutta-percha cone was inserted, the coronal end of the cone was trimmed using a heated plugger, cutting it 1 mm below the cemento-enamel junction. Any excess sealer was cleaned up with a damp cotton pellet and distilled water. To create a bacterial reservoir, two millimeters of gutta-percha were removed from the coronal part of the obturated root canals.

Following root canal filling, the specimens were radiographed to confirm adequacy of the obturation. After root canal obturation, the specimens were placed in an incubator with 100% humidity at 37 °C for one week to allow the sealer to set completely [23]. After incubation, the roots were apically resected perpendicular to the long axis of the root with a surgical bur positioned 3 mm from the apex, while being cooled with water using a surgical bur (SS White Zekrya Bur) [24].

The following steps were then performed for the three experimental groups using a dental operating microscope (Karl Kaps, Germany) at 10x magnification:

Group1: Roots were only resected without any further retrograde preparation and filling (SCT/ root resection).

Group2: Roots were resected, retroprepared and filled with calcium silicate sealer (NeoSEALER Flo; Avalon Biomed, Houston, TX, USA) (CaSi/ retrofilling).

After root-end resection, a 3-mm-deep retrograde cavity was meticulously prepared using a diamond-coated ultrasonic retro-tip (NSK VarioSurg3 Retrograde Endo Tip E32D-S) under a continuous sterile water spray and activated by a piezoelectric source (Newtron P5, Satelec Acteon) set at a power level of 6. The retro-prepared cavity was irrigated with sterile saline and dried using three sterile paper points. Subsequently, the calcium silicate-based sealer (NeoSealer Flo) was injected as a retrograde filling using a needle tip (Flex Flo tip) provided by the manufacturer.

After carefully positioning the tip in contact with the root-end cavity floor (gutta-percha filling), the sealer was injected into the retrograde cavity, and any excess was removed using a microbrush (FGM, Joinville, SC, Brazil) to ensure proper sealer adaptation.

Group 3: Roots were resected, retro-prepared, and filled with mineral trioxide aggregate. (MTA/ retrofilling).

The same retrograde preparation was performed as described in Group 2, followed by filling the 3-mm retrograde cavities with NeoMTA® 2 (Avalon Biomed, Houston, TX, USA). MTA was mixed following the manufacturer’s instructions and placed as increments using a carrier system (MAP System, Dentsply Tulsa, Tulsa, OK, USA) and compacted with an appropriately sized condenser.

In Group 2 and 3, periapical radiographs were taken to ensure the quality of retrograde filling. Subsequently, the specimens were placed in cryovials after being wrapped in pieces of sterile gauze moistened with phosphate-buffered saline (PBS) solution (pH = 8.4) [21]. All samples were stored in an incubator with 100% humidity at a temperature of 37º C for seven days to allow the retrofilling material to set completely [17, 25].

### For the control groups

Group 4 (positive control): Roots canals were filled only with a gutta-percha cone (size 40/0.04). Subsequently, root resection and retropreparation were performed, as mentioned earlier, without retrofilling.

Group 5 (negative control): Roots were resected and left unfilled, with no retropreparation or retrofilling without ensuing bacterial contamination.

### Preparation of bacterial leakage model

To prevent any leakage from possible lateral or accessory canals, the entire root surfaces in three experimental groups and positive control group were covered with two layers of nail varnish (Revlon Inc., New York, NY, USA), leaving only the resected surface uncovered. In the negative control group, both the entire root surfaces and resected surfaces were coated with two layers of nail varnish and utility wax.

The bacterial leakage model was conducted following the method described by Yanpiset et al. [20]. The microbial leakage model consisted of upper and lower chambers. The upper chamber was constructed using 1.5 mL sterile Eppendorf tubes (disposable scintillation vials; Sigma- Aldrich, St. Louis, MO).

Each specimen was inserted and stabilized into the cut bottom of the tube until approximately 8 mm of the specimen protruded through the cut end. All the interfaces between the tube wall and the specimen were completely sealed using cyanoacrylate adhesive (3 M Super Glue Gel, 3 M Company, Maplewood, MN, USA), followed by nail polish. The entire upper chamber model underwent ethylene oxide sterilization.

**Fig. 2:**
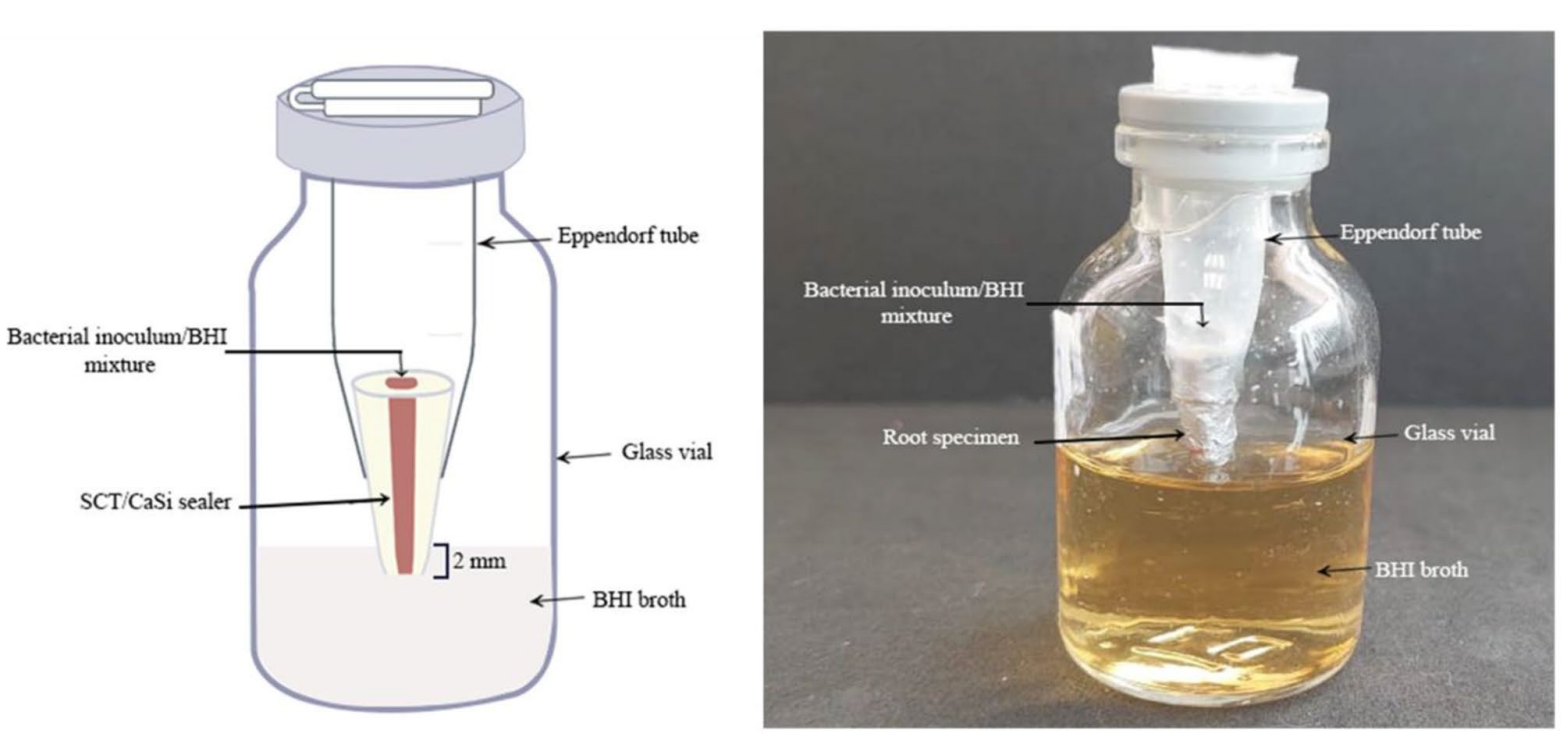
Bacterial leakage model. *E. faecalis*, *Enterococcus faecalis*; BHI, brain heart infusion; SCT, single cone technique

The lower chamber was a rubber-capped glass vial (Sigma-Aldrich Co., St. Louis, MO, USA) filled with BHI broth (HiMedia Laboratories Pvt. Ltd, India) and sterilized in an autoclave (Steam Autoclave HV-110, Hirayama, Tokyo, Japan) for 20 min at 121 °C. Prior to testing, the upper chamber Eppendorf tube was placed into the glass vial through the opening of the rubber cap, fitting tightly inside the glass vial. This step was performed in an UV-sterilized biosafety cabinet class II (Lamil plus 13, Finland) under strict aseptic conditions. Approximately 2–3 mm of the resected root end of the specimen was immersed in BHI broth without contacting the bottom of glass vial (Fig. 2). The junction between the tube lid and the vial rubber cap was sealed with cyanoacrylate adhesive. To ensure the sterility of the model, the assembled models were further incubated at 37 °C for 48 h. During this incubation period, none of the specimens showed any turbidity or other signs of microbial growth of BHI broth.

### Preparation, inoculation of bacteria in the model

For bacterial leakage testing, *Enterococcus faecalis* (*E. faecalis*) (ATCC 29,212) was employed. To begin, 24-hour colonies of *E. faecalis* were isolated from BHI agar plates and suspended in 3 ml of sterile BHI broth. These suspensions were then grown aerobically overnight at 37 °C. The following day, 120 µL of the overnight bacterial culture was aseptically transferred to 20 ml of fresh BHI broth until the log phase was reached (approximately 4 h and an optical density of 1 at 600 nm). Once in the log phase, the cultures were adjusted to a 0.5 McFarland standard (1.5 × 10^8^ colony-forming units (CFUs)/mL). Subsequently, each specimen was coronally filled with 40 µL of this bacterial inoculum using a sterile insulin syringe. The entire experiment was conducted aseptically under aerobic conditions at 37 °C for 30 days to ensure thorough bacterial penetration into the root canal space.

To maintain bacterial viability, the bacterial inoculum/BHI mixture was freshly prepared and replaced every three days using sterile techniques. All experimental and positive control specimens were subjected to bacterial inoculation, while negative controls were inoculated with sterile BHI broth.

### Evaluation of microbial leakage with ***E. faecalis***

Throughout the microbiological procedures and microbial leakage evaluation, the microbiologist was blinded of the group allocations. Bacterial leakage was assessed every 24 h by monitoring the turbidity of the BHI broth in the lower chamber for up to 30 days after inoculation with *E. faecalis* within the root canals. The time-to-leakage (days) was determined and recorded for each specimen. A 100 µL of suspension broth from each leaked specimen was plated onto blood agar plates and incubated aerobically at 37 °C overnight. Bacterial growth was confirmed by the appearance of smooth, cream, or white colonies of *E. faecalis*. Colony forming units (CFUs) were then counted. To verify the purity of *E. faecalis*, gram staining and biochemical tests, including growth in 6.5% NaCl in BHI and bile esculin, were conducted.

After 30 days, 100 µl of suspension broth from non-leaked specimens was subcultured onto blood agar to confirm the absence of bacterial leakage.

### Confocal laser microscopy analysis

Following the bacterial leakage test, five specimens were randomly selected from each group. These specimens were fixed in a self-cure acrylic, and a 1 mm segment of the apical root end was cut vertically along the long axis of the root using a diamond saw (Micracut 150, Metkon® metallography, Turkey) while being cooled with sterile distilled water.

The prepared discs were stained using a Live and Dead Bacterial Viability kit (L-7012 Molecular Probes; Eugene, OR, USA), which consists of two separate vials of two-component dyes (propidium iodide and SYTO9 mixed 1:1) for staining the bacteria. The dyes had excitation/emission maxima of 490–635 nm for propidium iodide, which stained dead bacteria in red, and 480–500 nm for SYTO 9, which stained active bacteria in green [26]. Following staining, the specimens were examined by CLSM (Leica DMi8; Leica Microsystems CMS GmbH, Germany). Single-channel imaging was used to display green and red fluorescence separately, after which the two channels were merged using LAS X software (version 1.1.0.12420; Leica Microsystems CMS GmbH). CLSM images of the bacteria within infected dentinal tubules were captured at a resolution of 1024 × 1024 pixels, and the specimens were assessed using ×5 and ×10 lenses.

### Statistical analysis

Comparison of bacterial leakage was done using chi-squared test with Monte Carlo correction of the *p* value. Comparisons of time to leakage and bacterial counts were done using Kruskal Wallis test. Kaplan Meier survival analysis was performed to compare the time to leakage among the study groups. The significance level was set at *p* value < 0.05. Data analysis was performed using SPSS for Windows (Version 26.0, IBM Corp.).

## Results

### Bacterial leakage analysis

All positive control specimens showed turbidity within 24 h after bacterial inoculation while none of the negative controls showed sign of turbidity which presents no contamination throughout the experimental procedures.

**Fig. 3:**
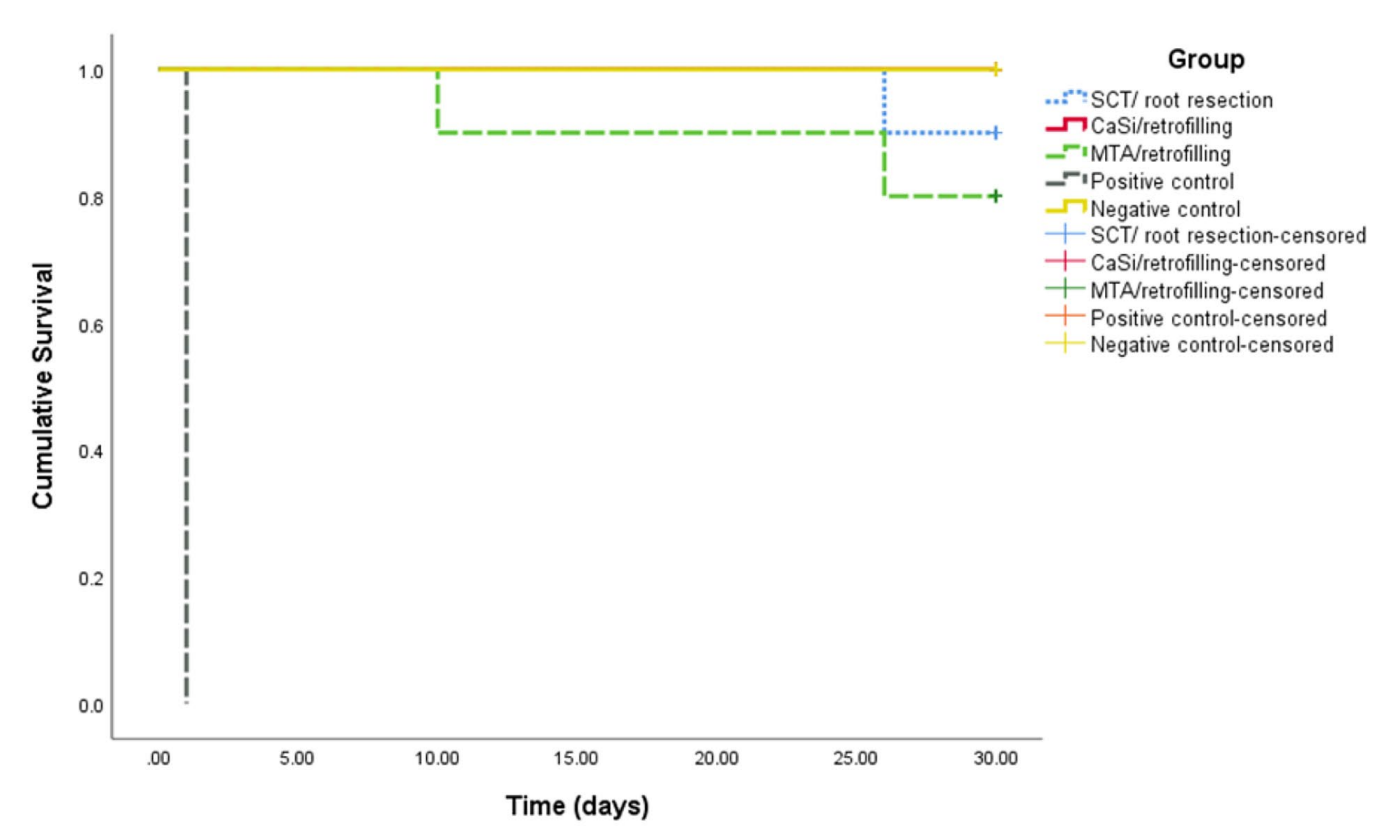
Kaplan-Meier survival curves of the five study groups

Table 1 shows the number, percentage of specimens that leaked or didn’t leak in the three experimental groups and the mean time to leakage after inoculation of E. faecalis for 30 days. The Kaplan-Meier survival curves of all study groups are presented in (Fig. 3). In the SCT /root resection group, only one specimen (10%) leaked at day 26 while no leakage occurred in the CaSi/ retrofilling group. In the MTA/retrofilling group, 2 specimens (20%) leaked at days 15 and 26 with mean time of 18.00 days.

**Fig. 4:**
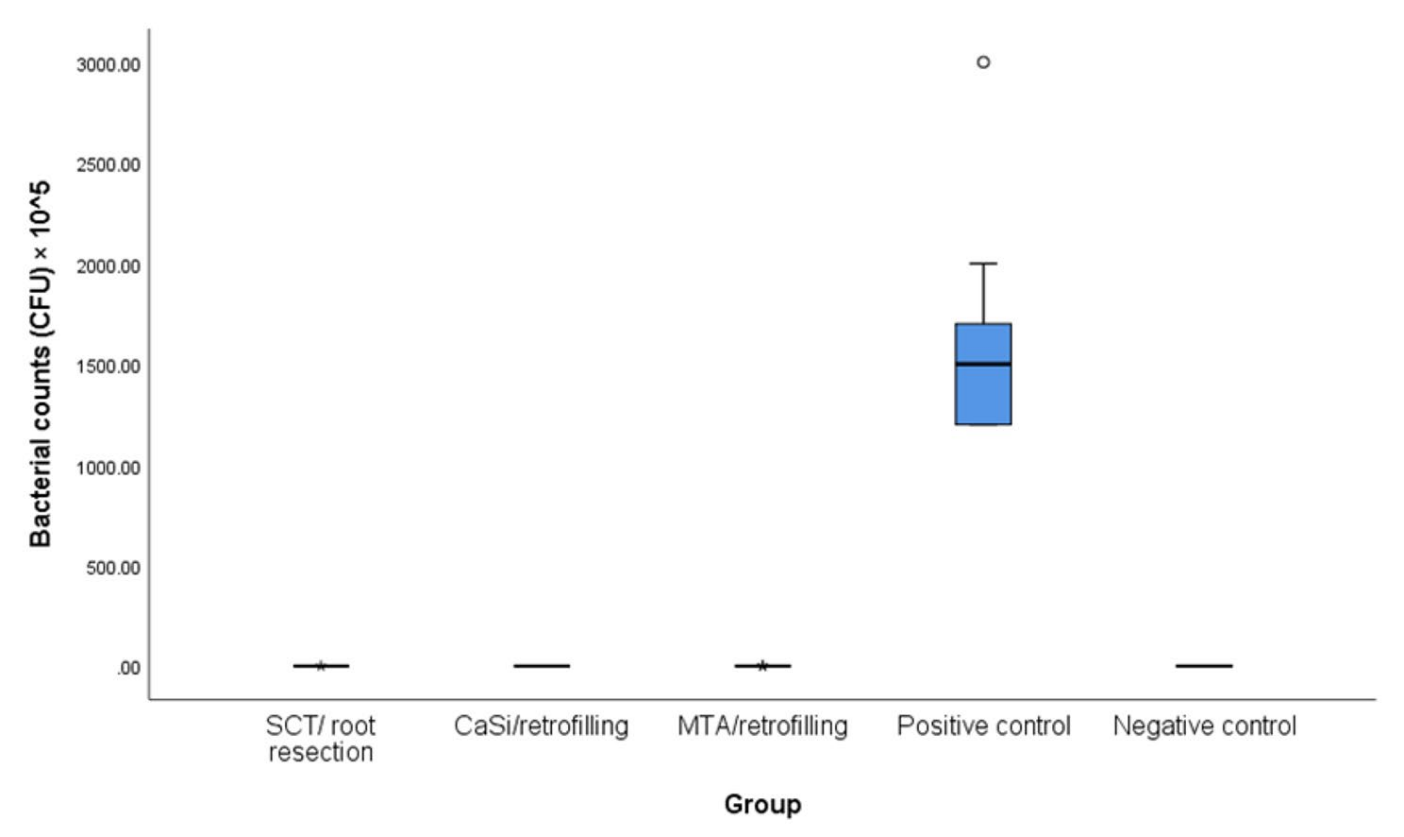
Bacterial counts (CFUs) in the study groups

The results revealed that SCT /root resection, CaSi/ retrofilling and MTA/retrofilling groups were not significantly different regarding bacterial leakage (*P* > 0.05) although CaSi/ retrofilling group exhibited the best sealing ability.

**Table 1:** Numbers (percentages) of specimens that leaked or did not leak and mean time to leakage in each experimental group (*n* = 10 each) after 30 days

Group	Specimens that leaked	Specimens that did not leak	Time to leakage: mean (SD)
	*N* (%)		
**SCT/root resection (n = 10)**	1 (10%)	9 (90%)	26.00 (0.00)
**CaSi/ retrofilling (n = 10)**	0 (0%)	10 (100%)	0.00 (0.00)
**MTA/retrofilling (n = 10)**	2 (20%)	8 (80%)	18.00 (11.31)
***P*** *value*	0.75		0.67

Values of ‘Time to leakage’ are presented as means ± standard deviations in days

SCT, single cone technique; CaSi- sealer, calcium silicate based sealer as retrograde filling; MTA, mineral trioxide aggregate

Bacterial counts (CFUs) of specimens for the five study groups are presented in Table 2 after 30 days of incubation. Positive bacterial growth was found in all specimens from positive control group with a mean number of 1.61 (0.55) × 10^8^(CFUs)/ml. No positive growth was recovered from specimens of the negative control group.

In MTA/retrofilling group, the mean counts of (CFUs)/ml was 0.27 (0.57) × 10^5^ and the mean counts of (CFUs)/ml was 0.10 (0.32) × 10^5^ for the group of SCT /root resection. While all specimens of CaSi/ retrofilling group didn’t have any colonies on the blood agar plates as this group completely resisted bacterial leakage.

There were no statistically significant differences in bacterial counts among the three experimental groups. Nevertheless, significant differences were observed when comparing the experimental groups to the positive control group. CaSi/ retrofilling group showed the same result as the negative control group.

**Table 2:** Bacterial counts (CFUs) in the five study groups after 30 days inoculation of Enterococcus faecalis

	SCT/root resection group(*n* = 10)	CaSi/ retrofilling (*n* = 10)	MTA/ retrofilling group (*n* = 10)	Positive control group (*n* = 10)	Negative control group(*n* = 10)	KWT *P* value
**Mean (SD)**	0.10 (0.32) × 10^5^**a**	0.00 (0.00) **a**	0.27 (0.57) × 10^5^**a**	1.61 (0.55) × 10^8^**b**	0.00 (0.00) **b**	**Z = 40.41**
**Median (IQR)**	0.00 (0.00)	0.00 (0.00)	0.00 (0.30) × 10^5^	1.50 (0.58) × 10^8^	0.00 (0.00)	P < 0.001*

KWT: Kruskal Wallis test, SD: Standard Deviation, IQR: Interquartile Range

*statistically significant at *p* value < 0.05

a.b: different letters denote significant differences between groups using Bonferroni correction

### Descriptive analysis of Confocal Laser microscopic images

The CLSM (×5 and ×10 magnification) images revealed viable bacterial penetration in all the positive control group specimens by the green fluorescence. Meanwhile all the negative control group specimens showed no fluorescence because of the absolute absence of bacteria.

**Fig. 5:**
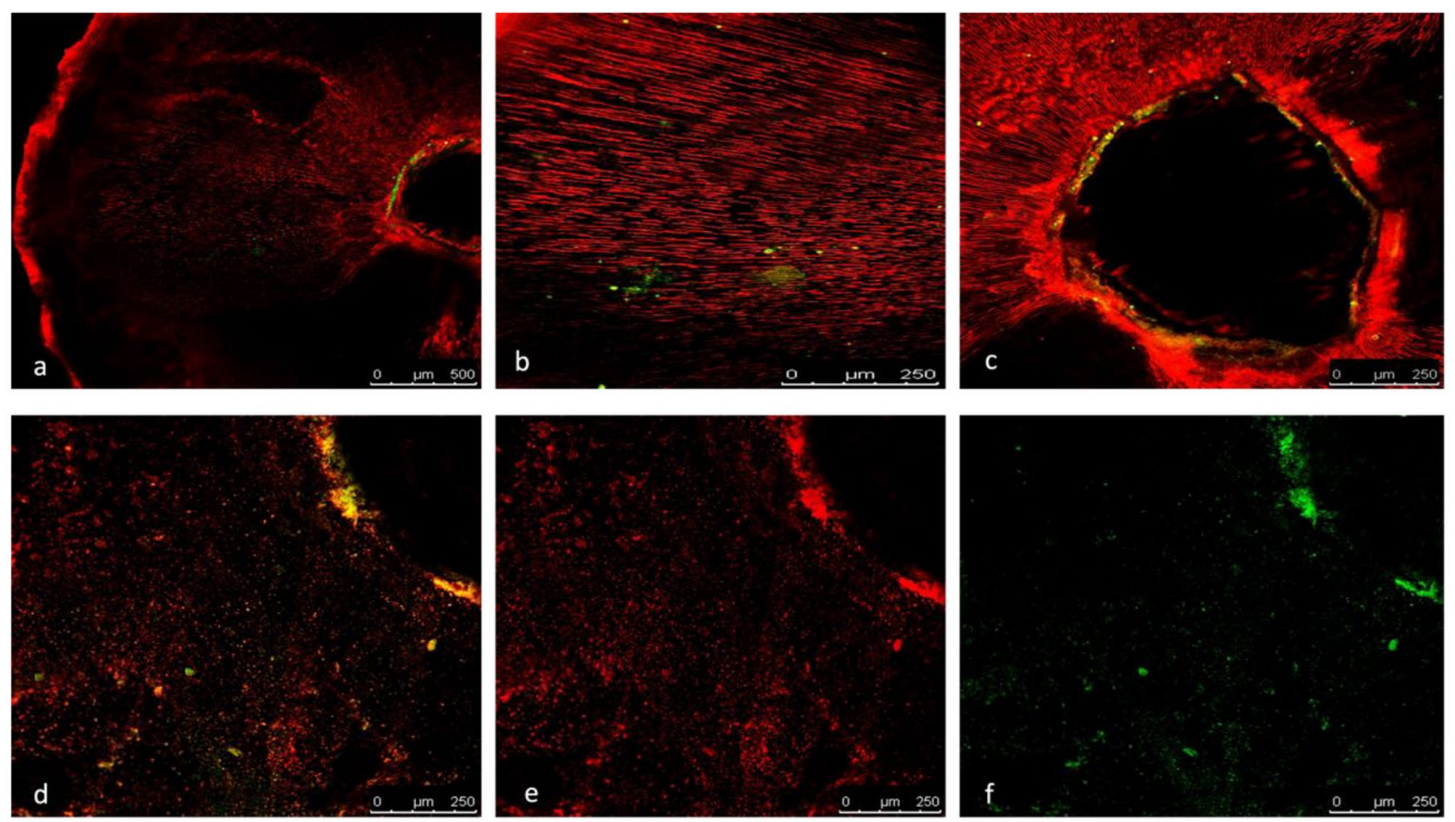
(**a-f**): demonstrating images from confocal laser scanning microscopy (CLSM) (magnification scale; a: 0–500 μm, b–f: 0–250 μm), of the E.fecalis bacterial penetration into the dentinal tubules of experimental group specimens .Green displays live bacteria(f), red displays dead bacteria (e).The bacteria can be presented at high (b-f) and low magnification (a)

**Fig. 6:**
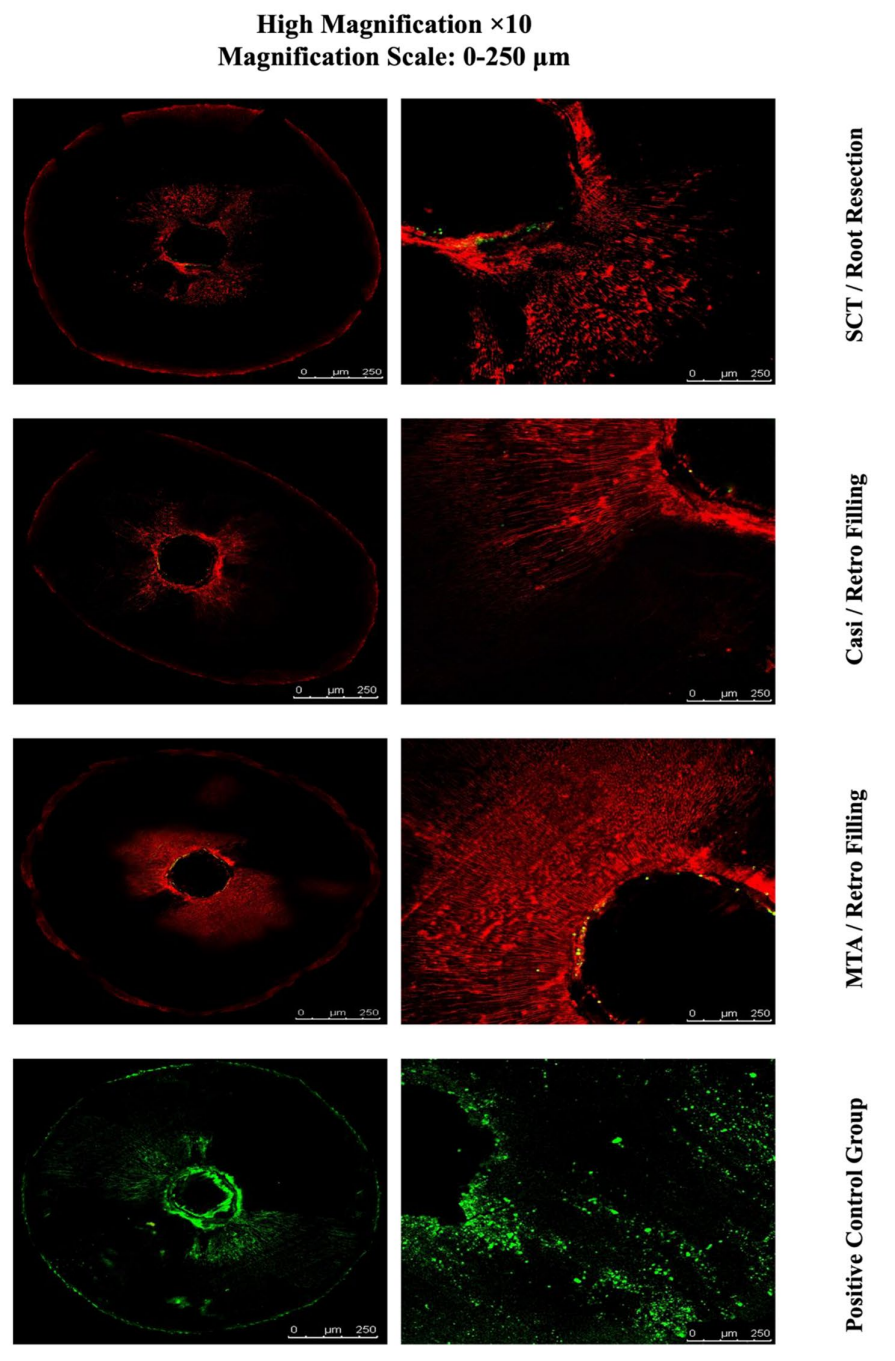
Images of CLSM demonstrating the detected fluorescence of the three experimental groups and positive control group. Dead (red) and live (green) bacteria penetrated the dentinal tubules

For the three experimental groups, the red fluorescence indicating dead bacteria was the prominent feature seen with no visible difference in the distribution of the dead bacteria. Although minute spots of green fluorescence where detected nearby the root canal lumen (Figs. 5 and 6).

## Discussion

In view of evolution of calcium silicate-based sealers and in order to simplify endodontic surgical procedures, the present study was designed to compare the sealing ability of calcium silicate-based sealer with SCT, followed by root end resection only and calcium silicate-based sealer as a retrograde filling versus MTA retrofilling within a bacterial leakage model.

To the best of our knowledge, this study is the first to address the microbiological perspective of calcium silicate-based sealer used with the SCT, followed by root end resection only. Additionally, it is the first to investigate the use of calcium silicate-based sealer as a retrograde filling material. The current results indicated no significant differences in bacterial sealing among the experimental groups, although CaSi/ retrofilling group showed the best results; accordingly, the null hypothesis was accepted.

The results of the bacterial sealing ability and CLSM findings were consistent, with CLSM images indicating viable bacterial penetration in all positive control group specimens, while the experimental groups showed predominantly dead bacteria.

Bacterial leakage is widely recognized as the primary contributor to apical periodontitis and treatment failure [26]. Therefore, this study employed a bacterial leakage model that utilized viable bacteria as indicators, making it a more clinically and biologically pertinent approach [17, 27]. Furthermore, this technique is commonly utilized for evaluating the efficacy of root end filling materials, offering precise and consistent data [23]. It is worth noting that the technique has its potential limitations, as it may permit leakage through different pathways aside from the root canal space [16, 25]. In order to overcome the drawbacks of this model, the routes of bacterial leakage through root filled teeth have been confirmed histologically using CLSM, resulting in more reliable results [25, 28]. The negative control specimens showed no turbidity or bacterial fluorescence for 30 days, while all positive control specimens showed turbidity and fluorescence, ensuring the accuracy of the leakage model with proper histological control groups.

Teeth with initial apical diameter size 25 were only included in order to standardize a size 40 final apical diameter [29] as this diameter would provide the circular shape of the prepared canal [20] for better adaptation with the round matched cone.

As the hydration process of calcium silicate-based materials can be impeded by a concluding rinse with NaOCl or EDTA, a final rinse with saline was executed to eliminate any lingering chemical residues from these substances [21]. Additionally, only three paper points were used to dry the canals before obturation. This was done to maintain a slight moisture content within the dentin inside the canals, which is essential for the proper hydration of the calcium silicate sealer [20, 21, 30].

E. faecalis is regarded as a suitable model for evaluating the effectiveness of root end filling materials [16, 17, 20, 27], so it was selected to be used in this study, as it is commonly linked with persistent apical periodontitis in root canals with dense fillings [31, 32].

In the current study, we assessed the application of SCT combined with root end resection without additional root end preparation. Several factors influenced this choice. This technique could result in greater apical root dentin thickness, potentially enhancing the overall outcome of endodontic surgery as reported by Ng and Gulabivala [33]. A previous study indicated that the use of ultrasonics for retrograde cavity preparation might lead to the development of microcracks in the apical dentin [34]. A clinical study with a long follow-up period of up to 6 years involving various cases that received MTA as a retrograde filling reported subsequent extractions due to vertical root fractures despite post-surgical healing [35]. Moreover, the SCT in combination with a calcium silicate-based sealer is considered more clinically practical, standardized, and reproducible. This approach is straightforward, easy to learn, simple to handle, and requires less time to complete compared to other root filling techniques [21, 36].

Based on the current findings, all three techniques demonstrated effective sealing against bacterial leakage. MTA is considered the gold standard for root end filling material. Thus, it was the reference technique in this study [37, 38], . Despite this, MTA has several limitations. In certain surgical scenarios,, the location of the surgical site and the small size of the root-end preparation can pose challenges when attempting to deliver MTA as a retrograde filling due to its granular and loose nature [39]. This can lead to increased porosity, unexpected voids, and potential microbial leakage [3, 37, 40].

The favorable outcomes for both the SCT/root resection and CaSi/ retrofilling groups in the current study could be attributed to the calcium silicate sealer’s ability to create a mineralized structure along the retrograde cavity margin that enhances the effective seal between the sealer and dentin interface, as previously reported [41]. Additionally, this sealer demonstrates low solubility and porosity, is highly hydrophilic, possesses a strong alkalizing capacity, and exhibits minimal expansion tendencies [12]. Notably, calcium silicate sealers also exhibit exceptional flowability, which allow them to penetrate dentinal tubules effectively [10]. This characteristic facilitates the filling of isthmuses and other complex apical anatomical features when used as a root end filling material.

Results of the present study align with Jung et al. [41], who observed comparable gap and internal void volumes in retrograde fillings using a combination of calcium silicate cement and calcium silicate-based sealer compared to a cement-only group [41]. Another previous study, utilizing an open apex model, demonstrated that applying a calcium silicate-based sealer to the canal walls before delivering calcium silicate-based cement orthogradely during apexification enhanced the cement’s adaptability to the root dentin wall [42].

A recent study highlighted that achieving deeper penetration of the retrograde filling hinders the bacteria’s ability to penetrate deeper into the dentinal tubules, consequently affecting their viability [28]. This finding aligns with our descriptive CLSM images analysis. Furthermore, the antibacterial properties of calcium silicate sealers may contribute to the elimination of any remaining microorganisms [43], thereby potentially enhancing the success rate of root end treatment.

It could be hypothesized that the excellent flow and proper adaptation of calcium silicate-based sealer in the critical apical region, which is essential in preventing bacterial recolonization, contributed to the effective sealing observed in the SCT with root end resection only. Some authors have reported a lower percentage of voids in the apical part of the root canal when using a calcium silicate-based sealer with the SCT [20, 21]. Moreover, the SCT is less likely to cause apical cracks as it applies less force to the tooth structure [9].

Recent studies [37, 44] have shown that the SCT with resection only resulted in the fewest voids compared to the standard retrograde approach. In the apical gutta-percha portion, no voids were detected, whereas voids were found in the MTA mass. Conversely, numerous studies have demonstrated that the outcomes of endodontic surgery are enhanced when a root end filling is applied, as opposed to merely smoothing the gutta-percha filling [35, 45]. It is worth noting that these studies may have used different types of sealers. However, calcium silicate-based sealers possess distinct physical and biological properties in comparison to previous sealer categories.

While all efforts were made to replicate clinical conditions, it is important to pinpoint the limitations of the present study. As an in vitro study, it may not encompass certain critical manipulative steps and clinical variables found in vivo, such as tooth type and position, tissue fluid, bleeding control, and the presence of periapical lesions.

Nonetheless, this study paves the way for future clinical research to investigate the long-term outcomes of employing SCT with root end resection only or CaSi retrofilling, comparing them to MTA to validate our findings. Moreover, there is a need to assess this novel technique using a multispecies biofilm model with relatively longer observation period to provide a more comprehensive understanding of its effectiveness. Additionally, expanding in vitro testing to include oval-shaped, irregular, and complex root canals would be valuable for a more thorough evaluation of this approach.

## Conclusions

Within the limitations of this study, it could be concluded that the bacterial sealing ability of calcium silicate-based sealer with the single cone technique combined with root end resection only and calcium silicate-based sealer as a retrograde filling were comparable with MTA retrofilling.

## Data Availability

The datasets used and/or analyzed in this study are available from the corresponding author upon reasonable request.
